# Supplementary material for: Gene Polymorphisms of NOD2, IL23R, PTPN2 and ATG16L1 in Patients with Crohn’s Disease: On the Way to Personalized Medicine?
Source: Genes (Basel). 2021 Jun 5;12(6):866. doi: 10.3390/genes12060866 (PMC8227795; doi:10.3390/genes12060866)
Supplement: Supplementary file 1 [file genes-12-00866-s001.zip › genes-1247304-supplementary.pdf]

Table S1. Baseline characteristics and therapy outcomes in CD patients according to NOD2 rs2066844, rs2066845 and rs2066847 polymorphisms

	NOD2 rs2066844 (n=338)			NOD2 rs2066845 (n=338)			NOD2 rs2066847 (n=338)		
**Variable**	**Wild type CC**	**Risk type CT and TT**	**P-value**	**Wild type GG**	**Risk type GC and CC**	**P-value**	**Wild type --**	**Risk type -C and CC**	**P-value**
n (%)	269 (79.6)	69 (20.4)		296 (87.6)	42 (12.4)		277 (82.0)	61 (18.0)	
Male, n (%)	120 (44.6)	34 (49.3)	0.488	130 (43.9)	24 (57.1)	0.107	124 (44.8)	30 (49.2)	0.531
Disease duration at baseline (years), mean ± SD	17.5 ± 11.3	18.8 ± 12.6	0.594	17.6 ± 11.7	19.0 ± 11.3	0.332	17.8 ± 11.8	17.7 ± 10.7	0.789
Age at diagnosis (years), mean ± SD	27.1 ± 10.9	26.7 ± 11.8	0.656	27.1 ± 10.9	26.3 ± 12.4	0.178	27.1 ± 10.9	26.9 ± 12.1	0.770
Montreal classification of IBD:									
Age, n (A1:A2:A3)	24:207:38	8:54:7	0.583	24:233:39	8:28:6	0.068	21:217:39	11:44:6	0.035
Phenotype, n (L1:L2:L3)	81:28:155 (n=264)	24:5:39 (n=68)	0.620	92:28:170 (n=290)	13:5:24	0.901	79:29:164 (n=272)	26:4:30 (n=60)	0.088
L4, n (%)	32 (12.1), n=264	7 (10.3), n=68	0.676	35 (12.1), n=290	4 (9.5)	0.800	33 (12.1), n=272	6 (10.0), n=60	0.642
Behavior, n (B1:B2:B3)	95:57:107, n=259	21:22:24, n=67	0.183	104:67:114 , n=285	12:12:17, n=41	0.596	94:63:110, n=267	22:16:21, n=59	0.710
Perianal disease, n (%)	75 (29.0), n=259	19 (28.4), n=67	0.923	81 (28.4), n=285	13 (31.7), n=41	0.664	82 (30.7), n=267	12 (20.3), n=59	0.111
First-degree relative(s) with IBD, n (%)	41 (16.1), n=255	13 (19.4), n=67	0.517	43 (15.3), n=281	11 (26.8), n=41	0.065	40 (15.2), n=263	14 (23.7), n=59	0.113
First-degree relative(s) with colon cancer, n (%)	11 (4.7), n=255	8 (12.7), n=63	0.020	18 (6.9), n=260	1 (2.6), n=39	0.485	15 (6.1), n=244	4 (7.3), n=55	0.761
Active or prior cigarette smoking, n (%)	81 (30.5), n=266	19 (27.9), n=68	0.687	84 (28.7), n=293	16 (39.0), n=41	0.175	86 (31.3), n=275	14 (23.7), n=59	0.251
Presence of at least one extraintestinal manifestation, n (%)	148 (55.0)	37 (53.6)	0.835	159 (53.7)	26 (61.9)	0.318	155 (56.0)	30 (49.2)	0.336
At least one prior IBD-related intestinal resection, n (%)	142 (52.8)	45 (65.2)	0.064	165 (55.7)	22 (52.4)	0.682	151 (54.5)	36 (59.0)	0.522
Prior IBD-related intestinal resections per disease year, median (IQR)	0.029 (0.090)	0.048 (0.110)	0.091	0.037 (0.090)	0.027 (0.090)	0.453	0.029 (0.090)	0.050 (0.090)	0.732

Azathioprine, n	138	31		142	27		135	34	
Response, n (%)	122 (88.4)	29 (93.5)	0.402	127 (89.4)	24 (88.9)	0.933	118 (87.4)	33 (97.1)	0.103
6-Mercaptopurine, n	7	2		8	1		7	2	
Response, n (%)	7 (100)	1 (50)	0.222	7 (87.5)	1 (100)	1.000	7 (100)	1 (50)	0.222
Methotrexate, n	40	6		38	8		43	3	
Response, n (%)	35 (87.5)	5 (83.3)	1.000	34 (89.5)	6 (75.0)	0.269	37 (86.0)	3 (100.0)	1.000
Infliximab, n	94	21		98	17		101	14	
Response, n (%)	83 (88.3)	20 (95.2)	0.347	86 (87.8)	17 (100.0)	0.127	90 (89.1)	13 (92.9)	0.667
Adalimumab, n	164	51		187	28		187	28	
Response, n (%)	150 (91.5)	45 (88.2)	0.488	168 (89.8)	27 (96.4)	0.263	170 (90.9)	25 (89.3)	0.783
Goliumumab, n	7	0		6	1		7	0	
Response, n (%)	5 (71.4)	0	-	4 (66.7)	1 (100)	1.000	5 (71.4)	0	-
Vedolizumab, n	64	13		68	9		66	11	
Response, n (%)	47 (73.4)	12 (92.3)	0.143	52 (76.5)	7 (77.8)	0.931	50 (75.8)	9 (81.8)	0.660
Ustekinumab, n	81	23		88	16		88	16	
Response, n (%)	69 (85.2)	18 (78.3)	0.428	73 (83.0)	14 (87.5)	0.651	72 (81.8)	15 (93.8)	0.235
Different prior IBD therapies from first diagnosis to baseline, n, median (IQR)	2.0 (2.0)	3.0 (3.0)	0.438	2.0 (3.0)	3.0 (3.0)	0.023	3.0 (2.0)	2.0 (2.0)	0.002
Number of different IBD therapies per disease year, median (IQR)	0.167 (0.220)	0.136 (0.200)	0.141	0.158 (0.220)	0.167 (0.250)	0.305	0.167 (0.250)	0.188 (0.200)	0.054
Prior exposure to therapies			0.504			0.126			0.002
0 therapy, n (%)	21 (7.8)	8 (11.6)		26 (8.8)	3 (7.1)		25 (9.0)	4 (6.6)	
1 therapy, n (%)	44 (16.4)	13 (18.8)		55 (18.6)	2 (4.8)		36 (13.0)	21 (34.4)	
2 therapies, n (%)	74 (27.5)	13 (18.8)		75 (25.3)	12 (28.6)		71 (25.6)	16 (26.2)	
3 therapies, n (%)	46 (17.1)	17 (24.6)		58 (19.6)	5 (11.9)		53 (19.1)	10 (16.4)	
4 therapies, n (%)	35 (13.0)	9 (13.0)		35 (11.8)	9 (21.4)		37 (13.4)	7 (11.5)	
5 therapies, n (%)	26 (9.7)	5 (7.2)		24 (8.1)	7 (16.7)		31 (11.2)	0 (0)	
6 therapies, n (%)	17 (6.3)	4 (5.8)		18 (6.1)	3 (7.1)		18 (6.5)	3 (4.9)	
7 therapies, n (%)	6 (2.2)	0 (0)		5 (1.7)	1 (2.4)		6 (2.2)	0 (0)	

CD: Crohn’s disease; IBD: inflammatory bowel disease; IQR: interquartile range; SD: standard deviation; TNFα: tumor necrosis factor alpha;
- no p-value calculated due to zero count in one of the categories

Table S2. Baseline characteristics and therapy outcomes in CD patients according to IL23R rs11209026, PTPN2 rs2542151, PTPN2 rs7234029 and ATG16L1 rs2241880 polymorphisms

	IL23R rs11209026 (n=376)			PTPN2 rs2542151 (n=373)			PTPN2 rs7234029 (n=373)			ATG16L1 rs2241880 (n=362)		
**Variable**	**Wild type GG**	**Risk type AG and AA**	**P-value**	**Wild type TT**	**Risk type TG and GG**	**P-value**	**Wild type AA**	**Risk type AG and GG**	**P-value**	**Wild type TT**	**Risk type TC and CC**	**P-value**
n (%)	351 (93.4)	25 (6.6)		260 (69.7)	113 (30.3)		258 (69.2)	115 (30.8)		64 (17.7)	298 (82.3)	
Male, n (%)	160 (45.6)	8 (32.0)	0.187	116 (44.6)	50 (44.2)	0.948	119 (46.1)	47 (40.9)	0.346	28 (43.8)	133 (44.6)	0.898
Disease duration at baseline (years), mean ± SD	17.4 ± 11.6	18.2 ± 10.8	0.573	17.4 ± 11.3	17.8 ± 12.0	0.874	17.7 ± 11.6	17.0 ± 11.3	0.670	17.3 ± 11.9	17.6 ± 11.6	0.744
Age at diagnosis (years), mean ± SD	27.6 ± 12.2	26.9 ± 7.4	0.395	27.5 ± 12.5	28.0 ± 10.6	0.180	27.3 ± 11.8	28.3 ± 12.1	0.391	28.7 ± 12.2	27.3 ± 11.7	0.323
Montreal classification of IBD:												
Age, n (A1:A2:A3)	34:261:56	2:22:1	0.241	30:189:41	6:91:16	0.139	28:194:36	8:86:21	0.330	4:49:11	30:226:42	0.558
Phenotype, n (L1:L2:L3)	108:40:198 (n=346)	7:2:15 (n=24)	0.842	83:28:146 (n=257)	32:13:65 (n=110)	0.827	82:24:148 (n=254)	33:17:63 (n=113)	0.284	14:12:37 (n=63)	99:26:168 (n=293)	0.027
L4, n (%)	45 (13.0), n=346	1 (4.2), n=24	0.336	32 (12.5), n=257	13 (11.8), n=110	0.865	35 (13.8), n=254	10 (8.8), n=113	0.184	9 (14.3), n=63	34 (11.6), n=293	0.553
Behaviour, n (B1:B2:B3)	127:85:127, n=339	7:4:13, n=24	0.261	84:67:99, n=250	48:22:40, n=110	0.155	83:64:100, n=247	49:25:39, n=113	0.204	23:15:24, n=62	103:71:113, n=287	0.984
Perianal disease, n (%)	95 (28.0), n=339	8 (33.3), n=24	0.577	70 (28.0), n=250	32 (29.1), n=110	0.832	67 (27.1), n=247	35 (31.0), n=113	0.452	22 (35.5), n=62	78 (27.2), n= 287	0.190

First-degree relative(s) with IBD, n (%)	58 (17.4), n=334	2 (8.3), n=24	0.395	43 (17.1), n=251	17 (16.2), n=105	0.829	41 (16.7), n=246	19 (17.3), n=110	0.888	7 (11.5), n=61	51 (18.0), n=284	0.219
First-degree relative(s) with colon cancer, n (%)	21 (6.8), n=311	1 (4.3), n=23	1.000	15 (6.4), n=236	7 (7.2), n=97	0.774	15 (6.5), n=232	7 (6.9), n=101	0.875	3 (5.1), n=59	18 (6.8), n=263	0.776
Active or prior cigarette smoking, n (%)	104 (30.1), n=346	4 (16.0)	0.173	74 (28.9), n=256	34 (30.4), n=112	0.779	70 (27.6), n=254	38 (33.3), n=114	0.261	21 (33.3), n=63	85 (28.9), n=294	0.486
Presence of at least one extraintestinal manifestation, n (%)	188 (53.6)	15 (60.0)	0.553	144 (55.4)	57 (50.4)	0.379	135 (52.3)	66 (57.4)	0.365	31 (48.4)	164 (55.0)	0.337
At least one prior IBD-related intestinal resection, n (%)	183 (52.1)	18 (72.0)	0.054	143 (55.0)	57 (50.4)	0.417	144 (55.8)	56 (48.7)	0.203	32 (50.0)	165 (55.4)	0.434
Prior IBD-related intestinal resections per disease year, median (IQR)	0.028 (0.090)	0.082 (0.120)	0.025	0.040 (0.100)	0.020 (0.080)	0.131	0.037 (0.100)	0.000 (0.070)	0.048	0.011 (0.090)	0.035 (0.090)	0.699
Azathioprine, n	180	11		129	62		128	63		34	151	
Response, n (%)	160 (88.9)	11 (100.0)	0.243	116 (89.9)	55 (88.7)	0.798	112 (87.5)	59 (93.7)	0.192	31 (91.2)	136 (90.1)	0.844
6-Mercaptopurine, n	9	1		9	1		10	0		2	7	
Response, n (%)	8 (88.9)	1 (100.0)	1.000	8 (88.9)	1 (100.0)	1.000	9 (90.0)	0	-	2 (100)	6 (85.7)	1.000
Methotrexate, n	49	4		35	16		40	11		5	44	
Response, n (%)	42 (85.7)	4 (100.0)	1.000	30 (85.7)	14 (87.5)	0.863	34 (85.0)	10 (90.9)	0.614	3 (60.0)	39 (88.6)	0.143
Infliximab, n	120	7		91	34		90	35		23	97	
Response, n (%)	108 (90.0)	7 (100.0)	0.379	84 (92.3)	29 (85.3)	0.236	81 (90.0)	32 (91.4)	0.808	21 (91.3)	88 (90.7)	0.931
Adalimumab, n	213	18		161	68		158	71		36	189	
Response, n (%)	194 (91.1)	17 (94.4)	0.626	146 (90.7)	63 (92.6)	0.631	144 (91.1)	65 (91.5)	0.919	33 (91.7)	173 (91.5)	0.979
Goliumumab, n	7	0		4	3		4	3		2	5	
Response, n (%)	5 (71.4)	0	-	3 (75.0)	2 (66.7)	1.000	4 (100.0)	1 (33.3)	0.143	1 (50.0)	4 (80.0)	1.000

Vedolizumab, n	77	5		55	27		61	21		14	67	
Response, n (%)	58 (75.3)	4 (80.0)	1.000	43 (78.2)	19 (70.4)	0.439	46 (75.4)	16 (76.2)	0.943	10 (71.4)	52 (77.6)	0.619
Ustekinumab, n	105	7		78	32		79	31		11	98	
Response, n (%)	88 (83.8)	6 (85.7)	0.894	67 (85.9)	25 (78.1)	0.317	71 (89.9)	21 (67.7)	0.005	9 (81.8)	82 (83.7)	0.875
Different prior IBD therapies from first diagnosis to baseline, n, median (IQR)	2.0 (3.0)	2.0 (2.5)	0.746	2.0 (3.0)	2.0 (3.0)	0.679	2.0 (3.0)	2.0 (3.0)	0.431	2.0 (2.75)	2.0 (3.0)	0.425
Number of different IBD therapies per disease year, median (IQR)	0.235 (0.240)	0.158 (0.200)	0.596	0.159 (0.260)	0.160 (0.190)	0.431	0.158 (0.240)	0.160 (0.180)	0.609	0.150 (0.260)	0.163 (0.220)	0.847
Prior exposure to therapies			0.868			0.741			0.738			0.357
0 therapy, n (%)	29 (8.3)	3 (12.0)		20 (7.7)	12 (10.6)		21 (8.1)	11 (9.6)		3 (4.7)	29 (9.7)	
1 therapy, n (%)	66 (18.8)	4 (16.0)		51 (19.6)	19 (16.8)		48 (18.6)	22 (19.1)		16 (25.0)	49 (16.4)	
2 therapies, n (%)	90 (25.6)	7 (28.0)		65 (25.0)	32 (28.3)		65 (25.2)	32 (27.8)		19 (29.7)	76 (25.5)	
3 therapies, n (%)	62 (17.7)	5 (20.0)		48 (18.5)	19 (16.8)		48 (18.6)	19 (16.5)		10 (15.6)	55 (18.5)	
4 therapies, n (%)	44 (12.5)	2 (8.0)		32 (12.3)	12 (10.6)		30 (11.6)	14 (12.2)		7 (10.9)	36 (12.1)	
5 therapies, n (%)	36 (10.3)	1 (4.0)		27 (10.4)	9 (8.0)		25 (9.7)	11 (6.6)		6 (9.4)	29 (9.7)	
6 therapies, n (%)	19 (5.4)	2 (8.0)		12 (4.6)	9 (8.0)		18 (7.0)	3 (2.6)		1 (1.6)	20 (9.7)	
7 therapies, n (%)	5 (1.4)	1 (4.0)		5 (1.9)	1 (0.9)		3 (1.2)	3 (2.6)		2 (3.1)	4 (1.3)	

CD: Crohn's disease; IBD: inflammatory bowel disease; IQR: interquartile range; SD: standard deviation; TNFα: tumour necrosis factor alpha;

- no p-value calculated due to zero count in one of the categories
